# Dual-guiding-layer resonance structure with an embedded metasurface for quasi-critical coupling without a perfect mirror

**DOI:** 10.1038/s41598-020-72983-8

**Published:** 2020-09-29

**Authors:** Gyeong Cheol Park, Kwangwook Park

**Affiliations:** 1 DTU Fotonik, Department of Photonics Engineering, Technical University of Denmark, 2800 Kgs. Lyngby, Denmark; 2 Electronics and Telecommunications Research Institute, Daejeon, 34129 Republic of Korea; 3 Division of Advanced Materials Engineering, Jeonbuk National University, Jeonju, 54896 Republic of Korea; 4 Hydrogen and Fuel Cell Research Center, Jeonbuk National University, Jeonju, 54896 Republic of Korea

**Keywords:** Optics and photonics, Optical materials and structures, Silicon photonics, Photonic devices

## Abstract

We propose an all-dielectric quasi-one-port resonance structure that achieves near perfect absorption without the use of a back mirror. The structure mainly consists of a high-refractive-index silicon metasurface and surrounding high-refractive-index guiding layers. The dual-guiding-layer (DGL) structure has high background reflectance and is designed to have a ratio of two decay rates into the upper and lower regions within a wider range. When an absorbing material is introduced into a DGL system, it can be designed to achieve a near critical-coupling condition by reducing the constraints in the two decay rates. By using single-layer graphene as an absorbing material, the DGL resonance structure shows an absorption of ~ 97% and a phase change of ∼ 0.95π near the wavelength of 1550 nm, confirming quasi-critical coupling. The optimized DGL structure is relatively insensitive to potential fabrication imperfections, and consequently, the expected average peak wavelength and absorption are obtained as 1549.29 nm and 96.74%, respectively. Angle-dependent absorption confirms that maximum absorption occurs under normal incidence. The DGL absorber is also designed to cover the whole *C*-band region, in order to meet the quasi-critical-coupling condition. All mode profiles are similarly quasi-symmetric along the metasurface due to the same DGL resonance mechanism.

## Introduction

Within the field of photonics, graphene has been extensively studied due to its gapless, fast electron mobility, and electro-optical properties. By integrating a graphene layer with a silicon (Si) or a silicon-on-insulator (SOI) platform, graphene-integrated silicon photonic devices have demonstrated their potential capabilities in high-speed operation, electro-optic modulation performance, and broadband light absorption^1–3^. In accordance with expansions in graphene-integrated silicon photonics, considerable efforts have been made to achieve direct transfer of wafer-scale graphene sheet onto a Si or a SOI platform^4^. This can also enable fabrication of graphene-integrated silicon photonic devices through a CMOS-compatible process in semiconductor foundries. For these reasons, graphene is regarded as an alternative to III–V materials or germanium as an absorbing material in silicon photonics^2,3,5^.

However, undoped single-layer graphene (SLG) suspended in air has a weak light absorption of ∼ 2.3% in the visible to near-infrared wavelength range^6^. To overcome its poor light absorption in waveguide-integrated photodetectors, a longer waveguide is needed, to enhance the light absorption path length. However, extending a waveguide, on the other hand, can possibly increase the scattering loss due to side-wall roughness of the waveguide. In addition, a lengthy waveguide decreases integration density of on-chip devices. To reduce the length of the waveguide, a planar resonance structure can be used on top of the graphene to enhance light-matter interaction^7,8^. In case of free-space light detection, an SLG can be transferred onto an SiO_2_ spacer layer, backed by a dielectric distributed Bragg reflector (DBR) to maximize the optical field in the graphene layer^9^. However, this still achieves only 9% of light absorption. Another structure that has been proposed is an optical cavity, formed of both upper and lower DBRs in order to achieve total absorption^5,10,11^. In this structure, the lower DBR ideally requires 100% reflectance and forms a one-port optical cavity. Total absorption can thus be achieved by controlling the upper DBR reflectance and the location of the graphene, although the poor heat dissipation caused by the presence of the DBRs is a potential drawback^12^.

As an alternative optical cavity, a guided resonance structure is used to meet the critical coupling condition, formed by a metasurface or 1D or 2D photonic crystal layer backed with a metallic or DBR^13–19^. The metasurface has a fixed thickness, but by manipulating the other parameters, such as grating period or the ratio of high to low refractive index regions within a unit cell, the decay rates of the resonance mode from the resonance structure can be tuned^13,20^. By manipulating these parameters and using a back mirror, the critical coupling condition can be met and thus total absorption is achieved^13,21–23^. To avoid the requirement of the back reflector, a dielectric photonic structure with inversion symmetry has been theoretically studied and proposed that achieves perfect absorption under off-normal incidence from one-side illumination^24^. However, in most cases, the inversion symmetry structure is too complicated to fabricate on an SOI platform.

Here, we propose and analyze a dual-guiding-layer (DGL) resonance structure on an SOI platform without using a back mirror, that achieves near-perfect absorption using single-layer graphene (SLG). The DGL structure shows high background reflectance and the ratio of the two decay rates into the incident and exit regions can be extended to a wider range. Consequently, the decay rate to the exit can be further suppressed and the structure becomes a quasi-one-port cavity. When an SLG is transferred onto the DGL structure, the sum of the two decay rates and the absorption rate of the SLG can be deliberately tuned so as to meet the quasi-critical-coupling condition. This DGL graphene absorber demonstrates ∼ 97.27% absorption and phase change of ∼ 0.95π under normal incidence at a wavelength of 1550 nm. The DGL structure is insensitive to potential fabrication deviation and covers the whole *C*-band region (1530–1565 nm). The design concept and its resonance mechanism can be applied to other photodetectors that use different material platforms, but without using a back mirror.

## Concept and design

**Figure 1:**
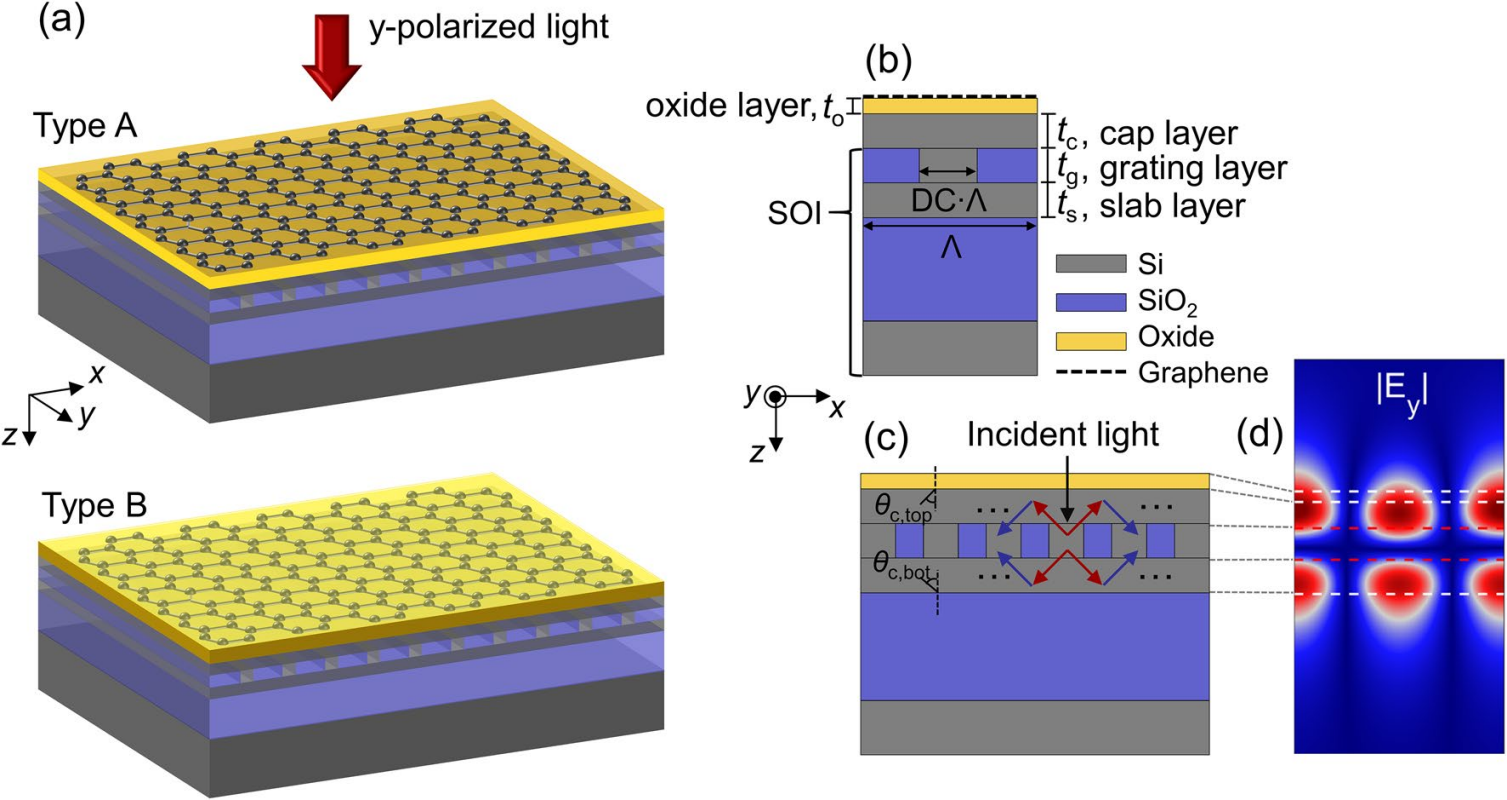
(**a**) DGL resonance absorbers on an SOI platform with an SLG on top of an oxide layer (Type A) and in-between the oxide layer (Type B). The incident light is transverse electric (TE) polarized (parallel to the grating bar). (**b**) Cross-sectional view of the DGL (Type A) with design parameters. Grating parameters: period (Λ), duty cycle (DC), grating bar width = (DC·Λ), grating thickness (*t*_g_), and thicknesses of three homogeneous layers: slab (*t*_s_), cap (*t*_c_), and oxide thickness (*t*_o_). (**c**) Schematic illustration of the physical mechanism of DGL resonance (red arrows: diffraction process; blue arrows: TIR). *θ*_c,top_: a critical angle for the top interface, *θ*_c,bot_: a critical angle for the bottom interface (graphene layer not included here). (**d**) Representative electric field amplitude: dotted lines correspond to different layers while red ones to the metasurface. The image is created by MathWorks MATLAB 2019b with in-house RCWA method.

Schematics for dual-guiding-layer (DGL) resonance photodetectors using an SLG as an absorbing material on an SOI platform are shown in Fig. 1a. An SLG is chosen since it is gapless and has almost constant absorption over a broad wavelength range^1,2^. Therefore, by tailoring the parameters of a DGL resonance structure, the absorption wavelength of a graphene-based DGL absorber can be tuned without replacing an absorbing material. However, it is worth to mention that other two-dimensional (2D) materials can be employed on a DGL platform as an absorbing material^2^. The SLG with no pattern can be transferred onto the top of the oxide layer (Type A) or sandwiched between the two oxide layers (Type B). An incident plane wave with transverse electric (TE) polarization is assumed. Unless otherwise stated, the incoming wave is incident with a normal angle. A cross-sectional view of the unit cell of the DGL resonance structure is shown in Fig. 1b. The structures mainly consist of three parts; cap and slab layers as a dual-guiding core, a metasurface to excite higher diffraction order(s), and an upper oxide layer. The thickness of the Si device layer is 250 nm. On the Si device layer, a metasurface layer (1D grating structure) is formed by partially etching down to the Si layer. Beneath the metasurface, an Si homogeneous layer remains, which becomes a slab layer. The spaces between the high-refractive-index Si grating bars are filled with the low-refractive-index material of SiO_2_ to form alternating Si and SiO_2_ bars in a lateral direction. On top of the metasurface, a high-refractive-index material such as amorphous Si (*a*-Si) is deposited as a cap layer, while a thin oxide layer with a low-refractive-index is deposited on top of the cap layer. The oxide layer can be aluminium oxide (Al_2_O_3_) or hafnium oxide (HfO_2_) to improve the quality of the transferred graphene and ameliorate contact resistance and electron mobility^25^. The proposed DGL structure is considered not only to design an optically optimized structure and to achieve near-perfect absorption, but also to consider the electrical performance of a graphene-based DGL absorber by adding a thin oxide layer^25^. The overall DGL structure is quasi-symmetric with respect to the metasurface, in terms of its composite optical refractive index. The first parameter, the grating period (Λ) of the metasurface, is determined in relation to the target wavelength and its diffraction angle. In considering the grating period, the initial excited diffraction orders from the normal incident light are restricted to ± 1st diffraction order. After the grating parameter has been established, the thicknesses of the slab and cap layers need to be optimized to enable the diffraction orders to propagate in the DGL structure, through the following diffraction process and total internal reflection (TIR). At a wavelength of 1550 nm, the diffracted angles in the cap and slab layers (both Si, *n*_si_ = 3.48) are calculated as ${\theta }_{\mathrm{m}}^{diff}=(\left|m\right|\times 1550\mathrm{ nm})/3.48/\Lambda$^26^. The grating period, which can excite the ± 1st diffraction order, should be larger than ∼ 445 nm but smaller than ∼ 890 nm in the Si layer. If the grating period is 800 nm, the ± 1st diffraction angle (${\theta }_{+1}^{diff}$) of both upper and lower guiding layers is ∼ 33.8°. At the upper oxide-cap interface, if use of HfO_2_ is assumed then the critical angle (${\theta }_{\mathrm{c},\mathrm{top}}$) is ∼ 36.5°. Even though the diffracted angle is slightly smaller than the upper critical angle, the TIR condition of the next oxide-air interface with a critical angle of ∼ 28.8° can reflect transmitted light back to the cap layer. At the lower slab-SiO_2_ interface, the lower critical angle (${\theta }_{\mathrm{c},\mathrm{bot}}$) is ∼ 24.6°. The diffracted light toward the lower boundary also experiences the TIR and is reflected back to the metasurface. The remaining two parameters to be determined are thickness and duty cycle (DC). The DC should be in the feasible range, such as from 0.3 to 0.7. Then, it can be fabricated using the deep ultraviolet (DUV) photolithography and dry etch process. The thickness of the metasurface should be determined so that it can tolerate possible fabrication imperfections. On top of the metasurface parameters, the thickness of both cap and oxide layers should be optimized to support resonance absorption. These parameters will be discussed in detail in “Results and discussion” section below.

A schematic description of the physical mechanism of the DGL resonance is shown in Fig. 1c. Normal incident light is transmitted and reaches the metasurface. The ± 1st diffraction orders are excited in the cap and slab layers and propagate toward the upper and lower homogeneous layers, indicated as red arrows. Since the homogeneous layers are composed of the high-refractive-index material of Si, the diffracted light at higher orders cannot penetrate the oxide-Si interface if it propagates at an angle greater than the critical angle at each interface. At the interface, the propagating light experiences TIR and is reflected back toward the metasurface, indicated as blue arrows. This in turn excites subsequent diffraction order(s) and the process is repeated. At the resonance condition, the DGL structure confines the light, as shown in Fig. 1d. The electric field profile is quasi-symmetric along the metasurface; this is because the resonance mode is initiated by the ± 1st diffraction orders in the upper Si cap and lower Si slab layer with the same diffraction angle amplitude (${\theta }_{+1}^{diff,cap/slab}=-{\theta }_{-1}^{diff,cap/slab}$). The subsequent process in the upper and lower guiding cores then also occurs symmetrically along the metasurface. The mode is extended outside the oxide and the BOX layer. The near field, which extended out of the top oxide layer, can interact with an SLG. Under optimum design parameters, a DGL resonant structure using an SLG can thus achieve near-perfect absorption, by closely matching up the decay rates from the DGL structure with the loss rate of the SLG.

## Numerical and theoretical analysis

To simulate DGL structures, an in-house rigorous-coupled wave analysis (RCWA) method is used^27^. For a two-port system, the resonance mode excited in a DGL resonator can be theoretically described by temporal coupled-mode theory (TCMT). The amplitude of the excited resonance mode *a* = *a*_0_*e*^*jωt*^ can be described as follows^20,28^:

1 $$\frac{da}{dt}=\left[j{w}_{0}-\left(\frac{1}{{\tau }_{1}}+\frac{1}{{\tau }_{2}}\right)-\frac{1}{{\tau }_{a}}\right]a+\left(\begin{array}{cc}{d}_{1}& {d}_{2}\end{array}\right)\left(\begin{array}{c}{S}_{+1}\\ {S}_{+2}\end{array}\right)$$

2 $$\left(\begin{array}{c}{S}_{1-}\\ {S}_{2-}\end{array}\right)={\mathrm{C}}\left(\begin{array}{c}{S}_{1+}\\ {S}_{2+}\end{array}\right)+a\left(\begin{array}{c}{d}_{1}\\ {d}_{2}\end{array}\right)$$

where *ω*_0_ is the resonance frequency; 1/*τ*_(1*,*2)_ (= *γ*_(1*,*2)_) is the decay rate of the amplitude of the mode into the two ports; 1/*τ*_*a*_ (= *γ*_*a*_) is the decay rate due to internal absorption; *S*_(1+*,*2+)_ and *S*_(1−,2−)_ are the incoming and outgoing plane waves; *d*_(1*,*2)_ is the coupling coefficient between the incoming waves and the resonance mode; and $$\mathrm{C}={e}^{j\pi }\left(\begin{array}{cc}r& jt\\ jt& r\end{array}\right)$$ describes the direct process through the reflection (*r*) and transmission (*t*) complex coefficients of the non-resonant interaction. S_2+_ is 0 when the structure is illuminated only from the top port. At the incoming and outgoing ports, the total wave is the sum of direct (‘background’) and indirect (‘resonance’) processes and the two processes can give rise to the Fano resonance^28^. If this is lossless (*τ*_*a*_* → *∞), it can conserve the total energy and is a time-reversal symmetry system^28^. In this system, the two decay rates, 1/*τ*_1_ and 1/*τ*_2_ are constrained by the direct process,

3 $$\frac{1-r}{1+r}\le \frac{{\tau }_{1}}{{\tau }_{2}}\le \frac{1+r}{1-r}$$

where *r* is the amplitude of the reflection coefficient of the direct process^28^. If a resonance system has a larger *r*, the ratio of the two decay rates is in a wider range. Importantly, within the bounds, even the asymmetric structure can have 100% reflectance^28^. This resonance structure can thus be regarded as a quasi-one-port resonance system without a back mirror around the resonance. If such a resonance system includes an absorbing material, the resonant process can be tuned so that the sum of the two decay rates into the two ports is similar to the internal absorption rate. It is thus possible to achieve quasi-critical coupling.

**Figure 2:**
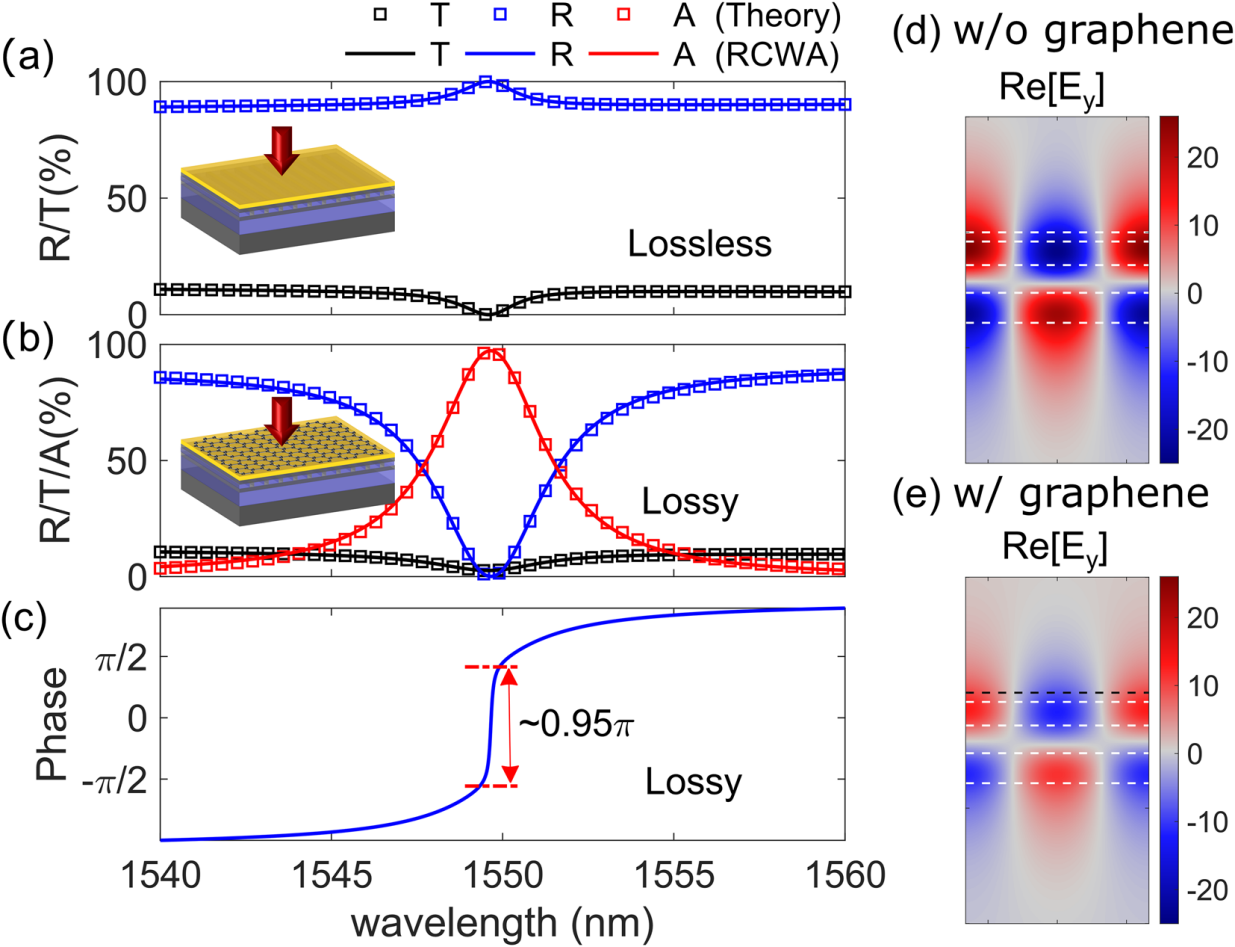
(**a**) Reflectance and transmittance spectra of the DGL resonance structure without SLG and (**d**) its electric field distribution; (**b**) reflectance, transmittance, and absorption spectra with SLG and (**e**) its electric field distribution; (**c**) phase spectrum of the reflection coefficient. Dotted lines in (**d**) and (**e**) indicate different layers of the DGL structure shown in Fig. 1 (black-dotted line: SLG). (**d**) and (**e**) are created by MathWorks MATLAB 2019b with in-house RCWA method.

The representative DGL structure on an SOI platform is analyzed. Figure 2a shows reflectance and transmittance spectra without an absorbing material; solid lines are obtained by RCWA simulation while square-dotted lines indicate the fitted results based on the TCMT^28,29^. The DGL structure’s high-refractive-index layers (Si) deliver high background reflectance, averaging as high as ∼ 89% within the wavelength range, as Fig. 2a shows. Average amplitude of the reflection coefficient is 0.943, while the corresponding upper and lower bounds of the decay rate are 34.0877 and 0.0293, respectively. By controlling the grating parameter, the two decay rates can be adjusted. The grating parameters of Λ and DC are 793 nm and 0.35, respectively. The thicknesses of the cap and HfO_2_ are 102 nm and 40 nm, respectively. Under these conditions, the Fano resonance is excited and 100% reflectance is achieved at 1549.59 nm, even though the DGL is an asymmetric structure^28^. From the theoretical fitting, the total decay rate (*γ*_*tot*_ = *γ*_1_ + *γ*_2_) is estimated as 0.7440 THz, and the decay probabilities (*η*_1*,*2_ = *γ*_1*,*2_/*γ*_*tot*_) of *η*_1_ and *η*_2_ are obtained as 0.956 and 0.044, respectively. The decay ratio is 21.72, which remains within the boundary.

On the same DGL structure, a graphene layer is placed as an absorbing material on top of the upper oxide layer. Figure 2b shows the corresponding reflectance, transmittance, and absorption spectra. The analytical TCMT (the square-dotted curve) is well fitted with the RCWA result (the solid line). The graphene’s complex refractive index enables it to absorb light; however, due to the imaginary part of its refractive index, the minimum transmittance for the two-port DGL system may increase^30^. Therefore, the maximum absorption of the DGL absorber using a graphene layer can be slightly less than 100% due to the intrinsic property of the two-port resonance structure, which does not use a back reflector. As shown in Fig. 2b, the minimum transmittance is 2.62%. The reflectance drops abruptly around the Fano resonance. The maximum absorption of the DGL graphene absorber is ∼ 97.27% at a wavelength of 1549.65 nm. The estimated total decay rate is 1.4832 THz, which is almost twice as large as that of the lossless DGL resonator. The decay probabilities (*η*_1_ and *η*_2_) into the two ports are 0.481 and 0.0132, respectively. The absorption decay probability (*η*_*a*_) is 0.5058. The DGL resonator is thus a slightly over-coupled system (*γ*_1_ + *γ*_2_ > *γ*_*a*_). The ratio of (*η*_1_ + *η*_2_)/*η*_*a*_ is 0.977 is close to the critical coupling condition of 1. The phase spectrum (argument of the reflection coefficient, ∠r) of the DGL resonator is shown in Fig. 2c. The phase change around the resonance wavelength is ∼ 0.95*π* with almost vertical slope. At the critical coupling condition, the phase change at the resonance wavelength is *π*^31^. This phase spectrum is another indication of a quasi-critical-coupling condition^31,32^. The electric field distribution at the resonance wavelength is shown in Fig. 2d,e, respectively, without and with an SLG. The field distribution shows an odd-like mode along the metasurface plane for both, and the evanescent field extends outside the upper and lower oxide boundaries. When a lossy material, such as an SLG, is transferred on top of the oxide, the peak electric field amplitude decreases by almost half, since the SLG absorbs the light. The overall field profile, however, is not significantly altered by the SLG. The property of an absorbing material determines *γ*_*a*_; however, *γ*_*a*_ is not determined solely by material properties, but also by the interaction with the cavity structure^20^. Since the field distribution and its overlap with the absorbing material will decide the *γ*_*a*_ for a Type B structure where an SLG is embedded inside the oxide layer, the DGL structure needs to be adjusted in order to achieve the near critical-coupling condition.

## Results and discussion

**Figure 3:**
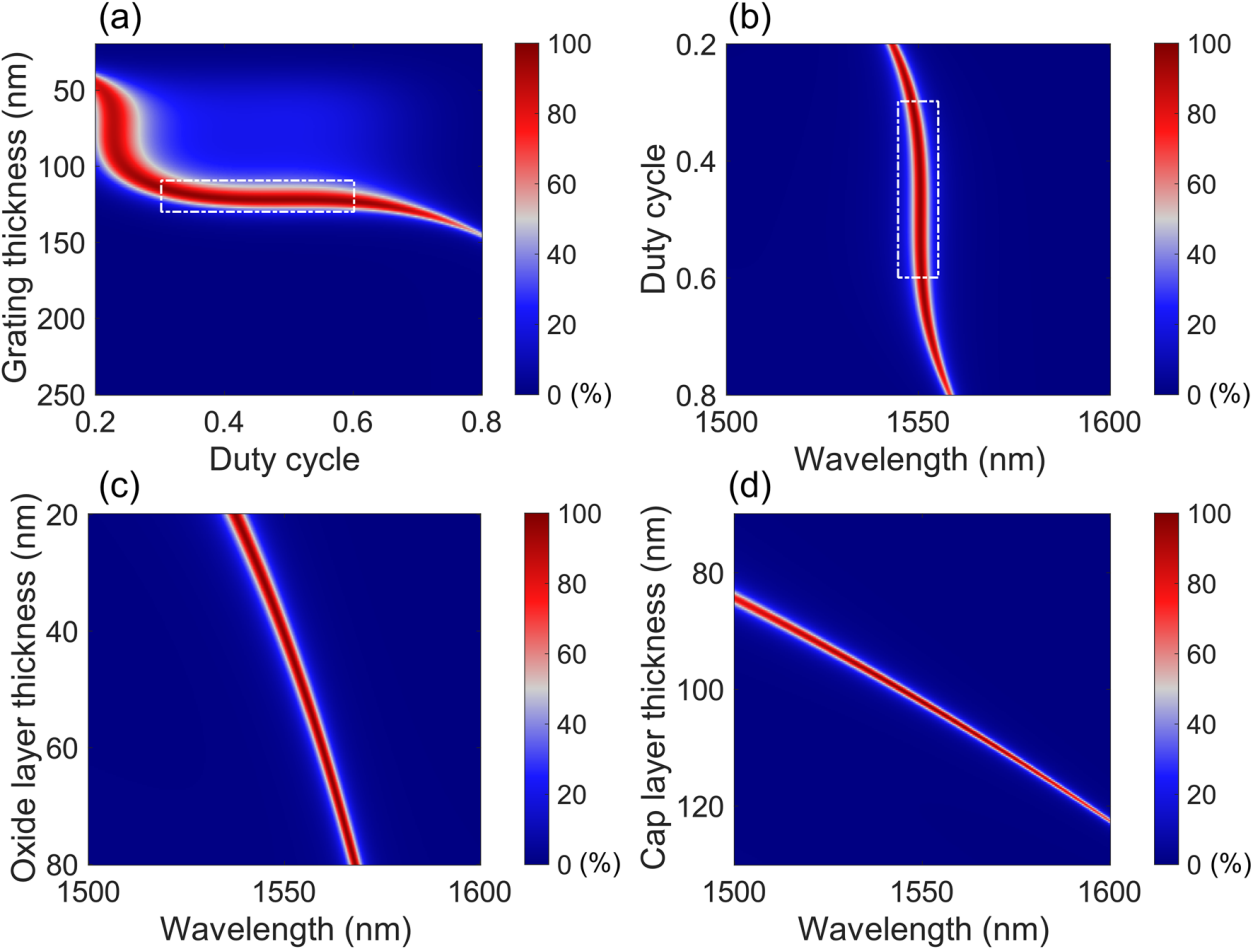
Absorption contour plots as a function of two grating parameters: (**a**) grating thickness versus duty cycle, (**b**) duty cycle versus wavelength. Absorption contour plots as a function of thickness of two homogeneous layers above the grating and wavelength: (**c**) oxide layer thickness versus wavelength and (**d**) cap layer thickness versus wavelength. The images are created by MathWorks MATLAB 2019b with in-house RCWA method.

To determine the optimum parametric set of a DGL structure and to analyze it, absorption is calculated by changing parameters. For the RCWA simulation, the graphene thickness is set as 0.335 nm and the complex refractive is estimated based on the equation of *n*_SLG_ = 3.0 + *j*(5.446/3 µm^−1^)*λ*, where *λ* is the wavelength^33^. The transferred SLG is assumed to be undoped. The refractive index of Si is assumed to be the same as 3.48. The refractive index of HfO_2_ and SiO_2_ are 2.07 and 1.45, respectively. Figure 3a shows the absorption map for a Type A DGL structure by changing grating parameters under normal incidence of TE polarized light with a wavelength of 1550 nm. By changing the DC and thickness of a grating, the absorption is calculated. The grating period of Λ, the thickness of an Si cap layer, and the thickness of HfO_2_ are 793, 102, and 40 nm, respectively, which are determined by the sub-optimization process. There is a near flat region of absorption of more than 95% around the grating thickness of 120 ± 10 nm, indicated by a white-dotted box. The duty cycle ranges from 0.3 to 0.6, which is converted to a grating bar width (DC·Λ) range of 238 to 476 nm. This grating can be fabricated using a DUV photolithography process. If the grating thickness is around 120 nm, the DC condition selected is flexible. Near the DC of 0.21, the shallow grating (less than 100 nm) region has a thick absorption region. However, the absorption is not as high as in the near flat region. The other absorption mapping is conducted by varying the DC and incident wavelength, as shown in Fig. 3b. The grating thickness is fixed as 120 nm. Around a target wavelength of 1550 nm and a DC range of 0.3 to 0.6, there is a region with more than 90% absorption inside the white-dotted box. Even outside the vertically flat region, there is a DC region where absorption is more than 90% within a slightly shorter or longer wavelength region. From the absorption maps in Fig. 3a,b, a DC of 0.35 and a grating thickness of 120 nm are selected. Even though the two parameters deviate from the target due to potential fabrication imperfections, the change of target wavelength can be minimized while maintaining peak absorption as high as possible.

The effect of oxide (HfO_2_) and Si cap layers on the absorption are investigated by varying its thickness and wavelength, as shown in Fig. 3c,d. The first layer to consider is the HfO_2_ layer. The grating period, DC, and thickness are fixed as 793 nm, 0.35, 120 nm, respectively. By increasing the thickness of HfO_2_ from 20 to 80 nm, the peak absorption wavelength is red-shifted from 1536 to 1564 nm, since the overall upper guiding structure (HfO_2_ + Si cap) becomes thicker and the supported resonance wavelength becomes longer. Within the thickness range of the HfO_2_, the average peak absorption is more than 96.98%. For the case of an Si cap layer with a 40 nm-thick HfO_2_, the peak absorption wavelength becomes longer, as does the HfO_2_ as the thickness of the Si cap increases, as shown in Fig. 3d. The average peak absorption of 95.69% is achieved within a thickness range from 97 to 107.5 nm, while the corresponding peak wavelength is distributed from 1536 to 1564 nm. Outside the thickness range of the Si cap, the DGL structure deviates from the quasi-critical-coupling condition, and hence the achievable peak absorption decreases. Since the refractive index of Si cap is higher than that of the oxide (HfO_2_), the peak wavelength shifts faster than it does for the oxide layer. Therefore, to adjust the peak wavelength precisely, control of the HfO_2_ is preferable. In addition, the atomic layer deposition (ALD) process itself is known for its precise control of deposition thickness^34^.

**Figure 4:**
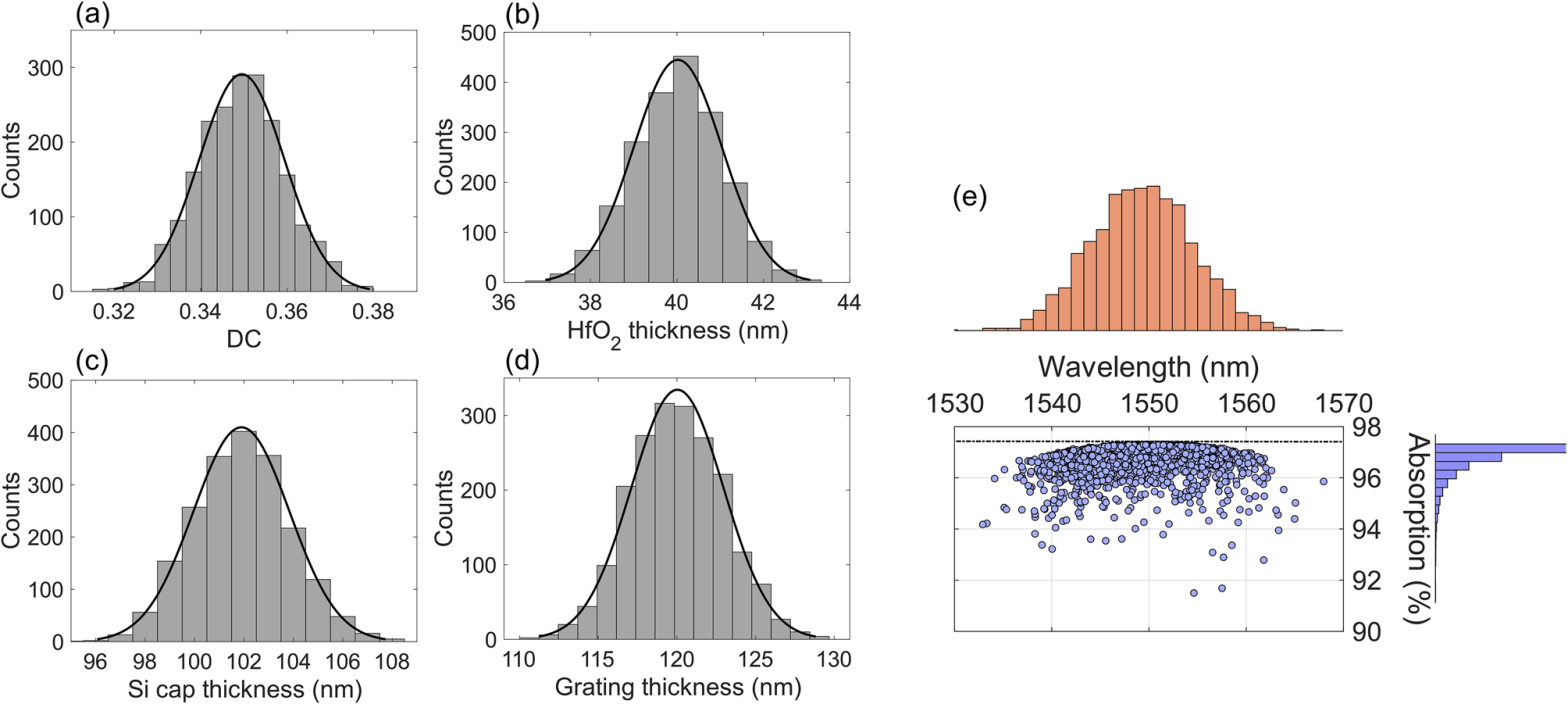
Estimation of peak absorption wavelength shift due to potential fabrication deviation, by randomly generating parametric spaces of (**a**) DC, (**b**) HfO_2_ thickness, (**c**) Si cap thickness, and (**d**) grating thickness of 2000 samples from a normal distribution. Black lines indicate the fitted normal distribution. (**e**) Scattering plot of absorption and corresponding peak wavelength shift (right inset: histogram of absorption distribution; top inset: histogram of peak wavelength shift from designed wavelength of 1550 nm).

The deviation from the design of the DGL structure can reduce peak absorption at the target wavelength because the resonance wavelength can be shifted, or may deviate from the quasi-critical-coupling condition. To investigate the design sensitivity of the DGL resonance structure, the main design parameters of grating thickness, DC, HfO_2_ thickness, and Si cap thickness are randomly generated following a normal distribution, $f\left(x\right)=\frac{1}{\sigma \sqrt{2\pi }}\mathrm{exp}\left[-\frac{1}{2}{\left(\frac{x-\mu }{\sigma }\right)}^{2}\right]$, where *µ* is the mean and *σ* is its standard deviation. The means of the parameters listed above are 120 nm, 0.35, 40 nm, 102 nm with the 3*σ* of 9 nm, 0.03, 3 nm, and 6 nm, respectively. The grating period is maintained as 793 nm. Each parameter is generated first with no correlation, then with these parameters, peak absorption and its wavelength are determined for 2000 samples. Figure 4a–d show the normal distribution for each parameter. The distribution of DC corresponds to variations in the grating bar width of ± 23.79 nm. The process for the HfO_2_ layer can be conducted using ALD within a variation in the ± 3 nm range. The black solid lines indicate the fitted normal distribution from the distribution of 2000 samples. Figure 4e shows the scattering plot of peak absorption and its corresponding wavelength. The average peak wavelength is 1549.29 nm, which is distributed within ± 20 nm. The average peak absorption is 96.74%, which is very close to the maximum absorption of 97.30%. The proposed design of the DGL graphene absorber thus remains robust within possible design imperfections, and can be feasible under optimization of the fabrication process.

**Figure 5:**
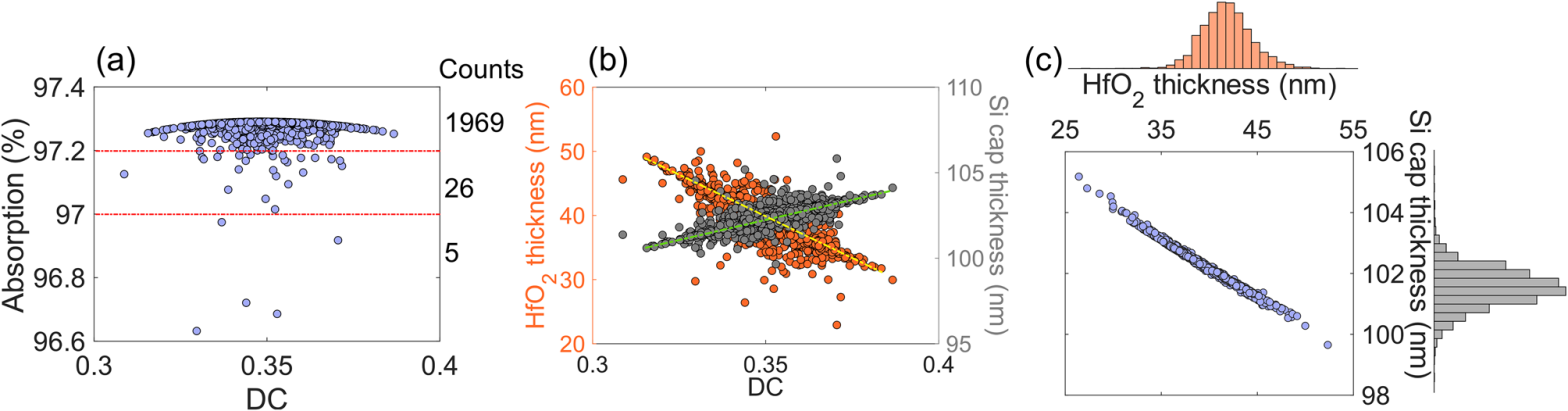
Thickness control of two homogeneous layers: HfO_2_ and Si cap, to maximize peak absorption at the target wavelength of 1550 nm when DC varies. (**a**) Maximum peak absorption distribution. (**b**) Thickness distribution of HfO_2_ and Si cap layer as a function of DC (orange dot: HfO_2_, green dot: Si cap). (**c**) Thickness correlation between HfO_2_ layer and Si cap.

The thickness of HfO_2_ and Si can be tuned in order to maximize peak absorption of the DGL graphene absorber at the target wavelength of 1550 nm. It is assumed that DC may deviate during fabrication, and DC values are randomly generated following normal distribution with *µ* = 0.35 and 3*σ* = 0.03. The grating thickness is set as 120 nm. These two optimum thicknesses are determined using particle swarm optimization (PSO)^35^. Figure 5a shows the distribution of peak absorption, with more than 1960 samples concentrated above 97.2%. Maximum peak absorption is 97.29% and minimum 96.63%. Average peak absorption is 97.27%. To analyze the correlation between the two homogeneous layers and the DC, a scattering graph is plotted as shown in Fig. 5b. As the DC increases, the overall thickness of Si cap increases (green dotted line), while the thickness of HfO_2_ decreases correspondingly (yellow dotted line). To confirm the correlation between the HfO_2_ layer and the Si cap, a scattered distribution is plotted as shown in Fig. 5c. On each axis, the thickness distribution of each homogeneous layer is plotted and follows the normal distribution. The HfO_2_ is mostly concentrated between 30 and 50 nm, while the Si cap is correspondingly between 101 and 104 nm. The correlation between the two layers is almost linear; the thicker the high-refractive-index Si cap layer, the thinner the low-refractive-index oxide layer. The additional controllability of the two homogeneous layers enables the DGL graphene absorber to achieve maximum absorption at the target wavelength.

**Figure 6:**
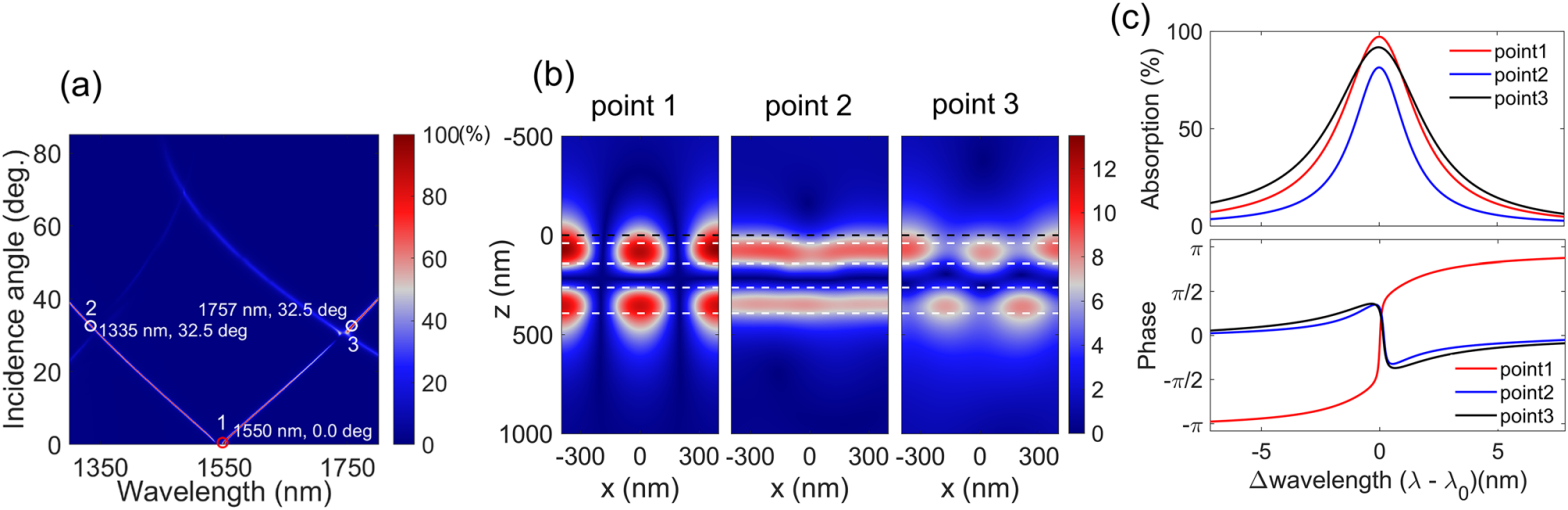
(**a**) Absorption spectra as a function of wavelength and incidence angle for Type A DGL structure. (**b**) Corresponding electric field (|E_y_|) profiles at the three points (dotted lines indicate different layers; black line indicates graphene position). (**c**) Absorption and phase (∠r) spectra, aligned with each peak wavelength. (**a**) and (**b**) are created by MathWorks MATLAB 2019b with in-house RCWA method.

The angle-dependent absorption for the DGL graphene absorber is shown in Fig. 6a. As the incidence angle increases, another absorption peak below the point 1 of 1550 nm appears and the two branches begin to diverge. Around 30°, another split in peak absorption occurs at wavelengths of 1340 and 1740 nm, respectively; the excited electric field profile (|E_*y*_|) at the corresponding three points is plotted in Fig. 6b. At the normal incidence case of point 1, the electric field profile is quasi-symmetric along the vertical axis because of the resonance mechanism in the DGL structure. However, as the incidence angle increases, the initial excited ± 1st diffraction orders are not symmetric along the surface normal, while the guiding layers of the DGL are not optimized to support the resonance process. As a result, the electric field profile is no longer quasi-symmetric along the surface normal, as shown in the respective field profiles of points 2 and 3 at the incidence angle of 32.5°. Moreover, the decay rates change and deviate from the quasi-critical-coupling condition. Therefore, peak absorption for the off-normal cases falls, as shown in Fig. 6c^13^. At the normal incidence case of point 1, peak absorption is 97.27%. At the respective off-normal cases of points 2 and 3, it decreases to 81.48% and 91.85%, respectively. From the phase spectra of Fig. 6c, at point 1, it is obvious that the phase changes abruptly, a further indication of the quasi-critical-coupling condition. At the higher incidence angle of points 2 and 3, it shows the properties of an under-coupled system^31,32^.

**Figure 7:**
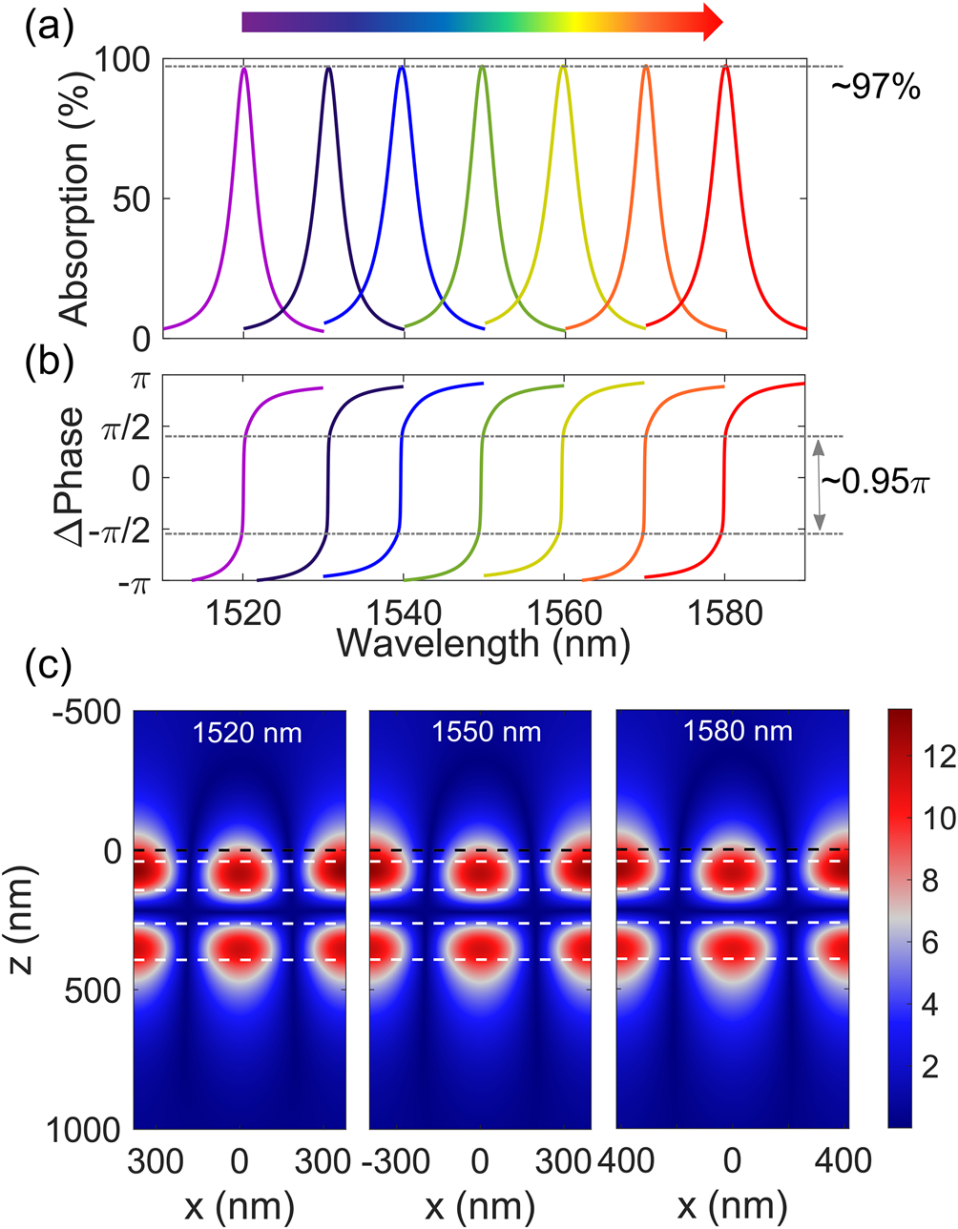
DGL graphene absorber with fixed 120 nm-thick grating for different peak wavelengths. (**a**) Absorption spectra of DGL graphene absorber designed for different peak absorption wavelengths. (**b**) Relative phase spectra of each DGL absorber. (**c**) Electric field amplitude distribution at respective peak wavelengths of 1520, 1550, and 1580 nm. The image is created by MathWorks MATLAB 2019b with in-house RCWA method.

The DGL graphene absorber covers the whole *C*-band wavelength range. The parameters of the DGL structure are determined using PSO with a fixed grating thickness of 120 nm, in order to achieve the quasi-critical coupling condition at the target wavelength. Figure 7a shows the exemplary absorption spectra of the DGL graphene absorber, designed for different wavelengths. From 1520 to 1580 nm, peak absorption at each design wavelength is ∼ 97%. As shown in Fig. 7b, an abrupt phase change of ∼ 0.95*π* is observed at each resonance wavelength, confirming quasi-critical coupling. The respective electric field amplitude profiles at wavelengths of 1520, 1550, and 1580 nm are shown in Fig. 7c; the field profiles for the three resonance wavelengths are quasi-symmetric along the z-axis and similar to each other. This is because of the resonance mechanism in the DGL structure, as explained earlier. On the SOI platform, the BOX layer separates the DGL structure from the lower handle Si wafer. Even though the BOX layer’s effect on absorption is not discussed here, there is a weak Fabry–Perot effect that slightly increases or decreases absorption depending on the resonance wavelength.

## Conclusion

In summary, we have proposed a dual-guiding-layer (DGL) graphene absorber on an SOI platform without any back reflector, which enables a near critical-coupling condition. Since the DGL structure has been designed to have a high background reflectance, it possessed the ratio of two decay rates of two-port system within a wider range. As a result, the sum of the two decay rates and the absorption rate caused by the graphene were closely matched. As a result, the ratio of the sum of the two decay rates to the absorption rate of 0.977 in the DGL resonance absorber was close to that of the critical coupling condition of 1. In addition, the phase change was as abrupt as ∼ 0.95*π*, a further indication of quasi-critical-coupling, and showed ∼ 97% absorption across the whole *C*-band range. Since the DGL resonance mechanism was identical for difference resonance wavelengths, the corresponding electric field profiles were similar. For angle-dependent absorption, maximum absorption was achieved at normal incidence. The principles underlying the DGL resonance system may thus be usefully applied to other resonance structures that use different material platforms and require a back mirror. On an SOI platform a DGL graphene absorber array can also be fabricated using a CMOS-compatible process for image sensing or other applications involving free-space communication.
